# Mapping Human Uterine Disorders Through Single-Cell Transcriptomics

**DOI:** 10.3390/cells14030156

**Published:** 2025-01-21

**Authors:** Sandra Boldu-Fernández, Carolina Lliberos, Carlos Simon, Aymara Mas

**Affiliations:** 1 Carlos Simón Foundation, INCLIVA Health Research Institute, 46010 Valencia, Spain; sboldu@incliva.es (S.B.-F.); carolina.lliberos@gmail.com (C.L.); csimon@fundacioncarlossimon.com (C.S.); 2 Department of Obstetrics and Gynecology, Universidad de Valencia, 46010 Valencia, Spain; 3 Department of Pediatrics, Obstetrics and Gynecology, Beth Israel Deaconess Medical Center, Harvard University, Boston, MA 02215, USA

**Keywords:** single-cell RNA sequencing, uterine disorders, endometriosis, endometrial cancer, adenomyosis, uterine fibroids/leiomyoma

## Abstract

Disruptions in uterine tissue function contribute to disorders such as endometriosis, adenomyosis, endometrial cancer, and fibroids, which all significantly impact health and fertility. Advances in transcriptomics, particularly single-cell RNA sequencing, have revolutionized uterine biological research by revealing the cellular heterogeneity and molecular mechanisms underlying disease states. Single-cell RNA sequencing and spatial transcriptomics have mapped endometrial and myometrial cellular landscapes, which helped to identify critical cell types, signaling pathways, and phase-specific dynamics. Said transcriptomic technologies also identified stromal and immune cell dysfunctions, such as fibroblast-to-myofibroblast transitions and impaired macrophage activity, which drive fibrosis, chronic inflammation, and lesion persistence in endometriosis. For endometrial cancer, scRNA-seq uncovered tumor microenvironmental complexities, identifying cancer-associated fibroblast subtypes and immune cell profiles contributing to progression and therapeutic resistance. Similarly, studies on adenomyosis highlighted disrupted signaling pathways, including Wnt and VEGF, and novel progenitor cell populations linked to tissue invasion and neuroinflammation, while single-cell approaches characterized smooth muscle and fibroblast subpopulations in uterine fibroids, elucidating their roles in extracellular matrix remodeling and signaling pathways like ERK and mTOR. Despite challenges such as scalability and reproducibility, single-cell transcriptomic approaches may have potential applications in biomarker discovery, therapeutic target identification, and personalized medicine in gynecological disorders.

## 1. Introduction

The uterus, a dynamic muscular organ that undergoes cyclical changes throughout a woman’s reproductive years, develops from the paramesonephric ducts at 9–10 weeks of gestation and matures to adapt to physiological processes such as pregnancy and menopause [1]. By 14 weeks, the uterus differentiates into two distinct layers: the primordial endometrium and the myometrium [2].

**Figure 1:**
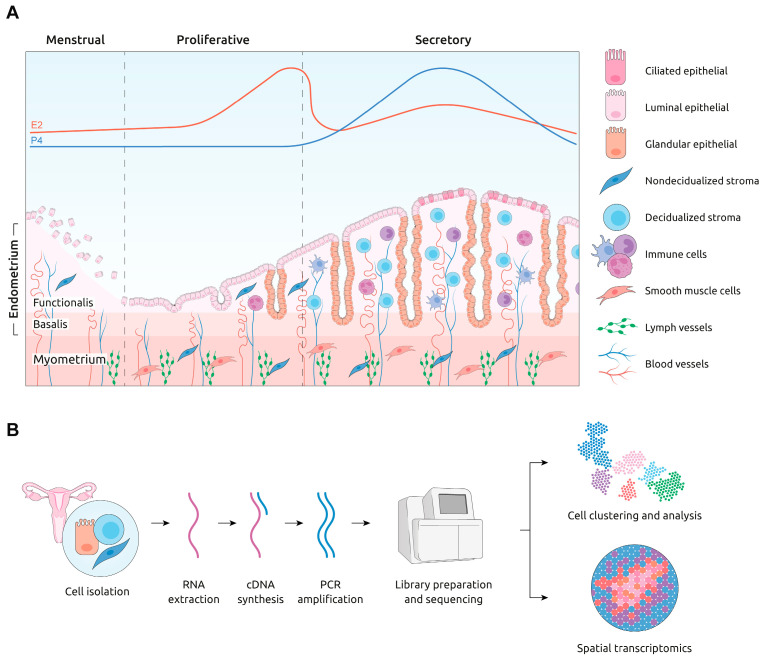
Illustration of (**A**) the human uterus showing the different layers and the morphological and cellular changes based on the phase of the menstrual cycle (i.e., menstrual, proliferative and secretory phase). Red and blue lines represent estradiol (E2) and progesterone (P4) fluctuations, respectively; (**B**) single-cell sequencing workflow. Briefly, the single-cell process involves the collection and dissociation of cells; extraction of total RNA, cDNA synthesis and PCR amplification; library preparation and subsequent sequencing; computational data analysis (expression profiling single-cell wise, clustering, and cell type identification, with each colored dot in the cell clusters representing a specific cell type, with each colored dot in the cell clusters representing a specific cell type).

The endometrium, the dynamic tissue lining the uterine cavity, is divided into two layers [3]. The *functionalis* layer contains various cell types, including epithelial, stromal, immune, and vascular cells, and it undergoes cyclic shedding, repair, and regeneration in response to ovarian hormones. The *basalis* layer, housing epithelial stem/progenitor cells, regenerates the *functionalis* after menstruation [4]. Below the endometrium lies the myometrium, which is a smooth muscle layer essential for menstruation and parturition regulated by hormones like progesterone, estrogen, and oxytocin [5,6,7]. Disruptions in these tissues can cause disorders affecting health and fertility, emphasizing the need to understand uterine biology for therapeutic advancements (Figure 1A).

Studies employing “bulk” transcriptomics have been pivotal in analyzing reproductive processes and identifying biomarkers for uterine disorders [8]. Microarray technology, widely used for uterine transcriptome analysis, helped to define the window of implantation to enhance assisted reproductive treatment outcomes [9,10] and aid the study of uterine disorders such as adenomyosis [11]. RNA sequencing (RNA-seq) surpasses microarray technology by offering higher resolution, greater sensitivity, reduced bias, and the ability to identify novel transcripts [12]. The application of RNA sequencing to studies of the endometrium and blastocyst revealed mechanisms affecting embryo implantation, improved assisted reproductive treatment outcomes [13,14], and advanced our understanding of gynecological disease [15,16,17]. While bulk RNA-seq results represent an average of gene expression values and, as such, mask cellular heterogeneity, single-cell (sc)RNA-seq addresses this limitation by profiling gene expression at the single-cell level, uncovering rare cell types and states [18]. Spatial transcriptomics further enhances these insights by integrating single-cell data with location within the tissue architecture, revealing cellular interactions and tissue organization [19]

ScRNA-seq experiments involve sample dissociation, individual cell isolation (using techniques such as microfluidics), and library construction for sequencing (Figure 1B) [19,20,21,22]. The subsequent analysis stage includes data preprocessing, quality control, dimensionality reduction, and cell type identification with optional additional analyses including cell communication or trajectory prediction [23]. Since being introduced by Tang et al. in 2009 [24], scRNA-seq has transformed reproductive medicine by elucidating the cellular compositions and gene expression profiles of healthy tissues and those affected by pathological uterine conditions [25,26,27,28,29,30,31].

This review highlights recent scRNA-seq-based studies of uterine disorders, which have shed light on the cellular interactions and mechanisms that significantly impact reproductive health and fertility.

## 2. Exploring Normal Uterine Function with Single-Cell Approaches

The comprehensive understanding of the cellular composition and molecular mechanisms at play in the human uterus remains an essential step toward unraveling the foundations of its normal functions and understanding the development of pathological conditions. This section highlights key findings that provide a basis for the advancement of knowledge regarding uterine biology.

The first scRNA-seq study of human endometrial tissue by Krjutskov et al. involved the isolation of single cells from two frozen endometrial biopsies, transcriptome sequencing, and subsequent data analysis, although it was limited to stromal and epithelial cells [32]. A more comprehensive study by Wang et al. followed, in which the authors characterized the human endometrium across the menstrual cycle [33]. Their analysis identified stromal fibroblasts, endothelial cells, macrophages, lymphocytes, unciliated epithelial cells, and a previously unknown cilium-associated epithelium and demonstrated robust correlations between cell type-specific transcriptomic profiles and the proliferative and secretory phases of the menstrual cycle. Subsequently, Garcia-Alonso et al. applied scRNA-seq and spatial transcriptomics to map cells of the endometrium, categorizing them into immune, epithelial (SOX9+, luminal, glandular, and ciliated), endothelial, stromal, and supporting (e.g., perivascular cells and smooth muscle cells [SMCs]) cells [34], which provided detailed insight into the spatial and temporal organization of these cell types within the human endometrium. Building on this work, Marečková et al. (from the same research group) further advanced the field by creating the most detailed reference atlas of the human endometrium to date [35]. They identified novel cell populations, including CDH2+ basalis cells with progenitor-like properties and unique epithelial and stromal cells present during the early secretory phase. They also described the spatiotemporal organization and functional dynamics of the endometrial epithelium throughout the menstrual cycle, significantly refining and expanding upon insights from earlier studies.

Single-cell approaches have also advanced the study of the human myometrium. Pique-Regi et al. developed the first single-cell atlas of the myometrium during term labor, revealing the cellular and molecular mechanisms involved in parturition [29]. Subsequently, Punzón-Jiménez et al. combined scRNA-seq with spatial transcriptomics to study myometrial aging in peri- and post-menopausal women, revealing the implication of novel cell types and aging-associated pathways [25]. Ji et al. provided a high-resolution comparison of non-pregnant and pregnant myometrial states, highlighting changes in SMC subpopulations and the role of M2 macrophages in pregnancy-associated anti-inflammatory responses [36]. Finally, Ulrich et al. integrated scRNA-seq datasets to create a consensus atlas of 39 uterine cell subtypes, detailing menstrual phase-specific changes and identifying a potential progenitor cell population [37].

In brief, these high-resolution “atlases” of uterine cellular and molecular landscapes provide the foundation for a deeper understanding of uterine biology and ongoing research into disorders such as endometriosis, endometrial cancer, adenomyosis, and fibroids.

## 3. Insights into Uterine Disorders from Single-Cell Studies

### 3.1. Endometriosis

Endometriosis, a condition characterized by the presence of endometrial-like tissue outside the uterine cavity, affects ~10% of women of reproductive age, impacting ~190 million women globally [38]. This chronic condition causes pelvic pain, dysmenorrhea, and infertility. It is also associated with an elevated risk of ovarian cancer [39,40] and is predominantly diagnosed through laparoscopy [41]. Endometriosis is widely attributed to retrograde menstruation with altered endometrial tissues transitioning into ectopic lesions [42]. Recent advances in single-cell transcriptomics have deepened our understanding of the cellular and molecular characteristics of endometriosis, identifying roles for stromal, epithelial, and immune cells in disease progression.

Ectopic and utopic endometrial tissues possess an increased proportion of fibroblasts with the elevated expression of genes such as DNA Topoisomerase II Alpha (*TOP2A*) and heightened anti-apoptotic signaling (e.g., through the MAPK pathway) promoting cell survival and proliferation. These fibroblasts also expressed osteoglycin and inflammation- and tumorigenesis-associated genes (i.e., *C3*, *C7*, and *S100A10*) [43,44]. However, their cellular origin remains controversial. While some studies have reported enhanced levels of fibroblast-to-myofibroblast transdifferentiation, which contributes to extracellular matrix (ECM) remodeling and fibrosis through TGF-β/Wnt signaling [42,45], others suggest that myofibroblasts in ectopic lesions may originate from endometrial mesenchymal stem cells (MSCs) under specific microenvironmental conditions [46]. Additionally, Zhang et al. demonstrated that activin A promotes the myofibroblast differentiation of endometrial MSCs through the STAT3-dependent Smad/connective tissue growth factor (CTGF) pathway [47].

Impaired decidualization, a process essential for pregnancy, further connects stromal cell dysfunction to inflammation, fibrosis, and senescence, exacerbating the condition [48,49,50]. Endometriotic epithelial cells exhibit disrupted homeostasis and apoptosis resistance [51] with the downregulation of *SULT1E1* leading to estradiol accumulation and enhanced MAPK signaling, which promotes survival and proliferation [44]. Emerging subpopulations of epithelial cells expressing the *SOX9* and *LGR5* stem cell markers have been implicated in endometriosis lesion growth with altered hormonal responses promoting ectopic proliferation during inappropriate menstrual phases [27]. Additionally, MUC5B+ epithelial cells display an elevated proliferative capacity, suggesting their role in establishing ectopic lesions [43].

Chronic inflammation also fosters a pro-tumorigenic microenvironment, potentially contributing to endometriosis-associated ovarian cancer (EAOC) [52]. Immune gene expression analysis showed similarities between some endometriosis patients and EAOC cases, including elevated *C3* and *C7* expression [53], in accordance with single-cell analyses [27,42,43]. Furthermore, Fonseca et al. applied multi-subject single-cell deconvolution across three independent datasets, revealing that clear cell and endometrioid ovarian cancers were enriched in ciliated endometrial-type epithelial cells, whereas high-grade serous ovarian cancers showed no epithelial cluster enrichment [27]. These findings support the hypothesis that endometrial-type epithelial cells may be precursors for clear cell and endometrioid ovarian cancers, linked to endometriosis, while high-grade serous ovarian cancers are likely unrelated [54].

**Figure 2:**
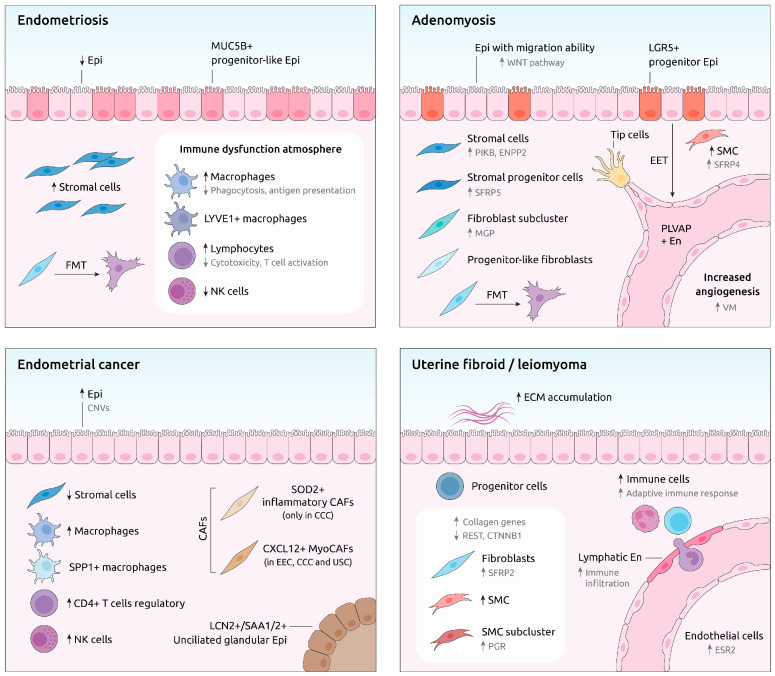
Representation of modified cell types, novel emerged cell types, key processes, and genes with altered expression under each condition compared to controls. Bold arrows indicate changes in cell abundance, while grey arrows denote changes in gene expression CAFs: cancer-associated fibroblasts; CCC: clear cell carcinoma; CNVs: copy number variations; Epi: epithelial cells; ECM: extracellular matrix; EEC: endometrioid endometrial cancer; EET: epithelial-to-endothelial transition; En: endothelial cells; FMT: fibroblast-to-myofibroblast transition; NK cells: natural killer cells; SMC: smooth muscle cell; USC: uterine serous carcinoma; VM: vasculogenic mimicry.

Immune dysfunction represents a pivotal mechanism in endometriosis pathology with myeloid cells (particularly macrophages) driving angiogenesis, tissue remodeling, and inflammation [43,55]. Myeloid cells display a diminished capacity for antigen presentation and phagocytosis in ectopic lesions, exacerbating chronic inflammation and lesion persistence [45,56]. Other immune alterations include reduced natural killer (NK) cell activity and impaired T-cell cytotoxicity, which further facilitate the survival of ectopic lesions [42,49]. The presence of lymphocytes and tertiary lymphoid structures in lesions underscores sustained immune dysregulation [43] (Figure 2).

In brief, dysregulated cellular signaling pathways, immune alterations, and hormonal imbalances drive fibrosis, chronic inflammation, and lesion persistence in endometriosis. Insight into these pathways, which include fibroblast-to-myofibroblast transdifferentiation, hormonal dysregulation, and immune impairment, provide targets for the development of novel treatment strategies.

### 3.2. Endometrial Cancer

Endometrial cancer (EC), a malignancy originating in the uterine epithelium, represents the second most prevalent gynecological cancer worldwide (after cervical cancer) with over 400,000 new cases and nearly 100,000 deaths reported in 2022 [57,58]. Early-stage diagnosis yields a five-year survival rate of ~81%, but this figure drastically declines to ~15% in advanced stages [59]. Interestingly, endometriosis represents a potential risk factor for EC [60]. Endometrioid endometrial cancer (EEC), the most common subtype (~85% of cases), is characterized by microsatellite instability and *PTEN*, *PIK3CA*, and *CTNNB1* gene mutations [61]. Despite a generally favorable prognosis, ~20% of EEC patients experience tumor recurrence, reducing the five-year survival rate from 81% to 10% [62]. Uterine serous carcinoma (USC), the second most common subtype (~10%), is frequently associated with *TP53* mutations [61], while rarer highly aggressive subtypes such as clear cell carcinoma (CCC) and uterine carcinosarcoma account for only ~5% of cases [63].

The EC tumor microenvironment exhibits significant heterogeneity with diverse cellular populations and genetic variability influencing disease progression [64]. Single-cell technologies facilitated the characterization of said complexities, revealing critical insights into tumor biology. Epithelial cell studies identified subclusters (e.g., stem-like, secretory glandular, and ciliated cells) that display chromosomal copy number variations and upregulated cancer-related gene expression [65,66]. Marker genes such as *LCN2* and *SAA1/2* have been implicated in EEC tumorigenesis, while subtypes like USC exhibit epithelial-to-mesenchymal transition, which supports metastasis [26,67]. Stromal fibroblasts, although reduced in number in EC compared to normal tissue, play a pivotal role thanks to the activity of cancer-associated fibroblasts (CAFs). CAF subtypes (including inflammatory, vascular, and antigen presenting) act to support tumorigenesis; indeed, the presence of SOD2+ iCAFs correlates with poor survival due to their metastasis-promoting role [26,68] (Figure 2).

Immune cells within the tumor microenvironment also contribute to EC progression and aid prognosis [69]. Specific tumor-associated macrophage (TAM) subtypes facilitate tumor growth and vascularization with their presence positively correlating with therapeutic responses [70,71]. T cells, particularly CD8+ tissue-resident memory T cells (TRMs), associate with a better prognosis, although immunosuppressive FOXP3+ T regulatory cells complicate the immune landscape [66,72]. Studies have highlighted the importance of NK cells and tertiary lymphoid structures, which represent favorable prognostic markers [73]. Integrating advanced profiling techniques has enhanced our understanding of critical pathways (e.g., PI3K/AKT/mTOR) and highlighted the potential for tailored therapies targeting specific tumor characteristics. These findings underscore the complexity of EC and the need for continued research to improve diagnostic and therapeutic strategies.

### 3.3. Adenomyosis

Adenomyosis is a benign gynecological condition characterized by the disruption of the endometrial–myometrial junction and the presence of ectopic endometrial tissue within the myometrium, which leads to myometrial hypertrophy and hyperplasia [74,75]. While some patients remain asymptomatic, most experience abnormal menstrual bleeding, dysmenorrhea, dyspareunia, chronic pelvic pain, or infertility [76]. Furthermore, an elevated risk of ovarian cancer has been identified in patients with adenomyosis; however, this relationship has been scarcely investigated, highlighting the need for additional studies to clarify their association [77,78]. The incidence of adenomyosis—estimated at 29 cases per 10,000 women annually—varies across studies due to population differences [79]. Although the precise pathogenesis of adenomyosis remains unclear, two primary hypotheses dominate: the invagination theory, which involves endometrial–myometrial junction injury and endometrial invasion into the myometrium, and the metaplasia hypothesis, which implicates progenitor cells undergoing dysregulated differentiation/migration [80,81]. Hormonal dysregulation, inflammation, angiogenesis, and fibrosis are also implicated, although the specific mechanisms remain incompletely understood [82,83].

Advances in single-cell technologies have significantly improved our understanding of adenomyosis by enabling a high-resolution analysis of cellular and molecular features within uterine tissues (Table 1). Studies have identified distinct populations of epithelial, stromal, endothelial, fibroblast, smooth muscle, and immune cells and the upregulated expression of genes involved in angiogenesis, cell growth, migration, and fibrosis (all associated with disease progression) [84,85]. While findings can diverge due to sample variability, these studies provide a deeper understanding of adenomyosis pathogenesis and highlight potential therapeutic targets.

Epithelial and stromal cells within the ectopic endometrium exhibit increased migratory and proliferative potential, supporting the invagination hypothesis of adenomyosis. These cells display alterations in Wnt signaling with genes such as *MMP7*, *CLDN4*, and *LGR5* playing significant roles in adenomyotic lesion development [85,86]. Fibroblasts and SMCs also possess multiple distinct subclusters in ectopic tissues, which associate with fibrosis, excessive ECM deposition, and SMC proliferation. The fibroblast-to-myofibroblast transition and Wnt signaling modulation by *SFRP* family genes further underscore the role of these cells in lesion formation and tissue remodeling [85,87]. Progenitor cells in adenomyotic tissues differentiate into lesion-specific stromal cells, further implicating dysregulated signaling pathways in adenomyosis progression [88,89]. Endothelial cells in adenomyotic tissues exhibit heightened angiogenic activity with the upregulation of *VEGF*, *ANGPT*, and other angiogenesis-related genes. Single-cell analyses showed evidence of vasculogenic mimicry [90] as well as the presence of endothelial tip cells [86], which can differentiate into various endothelial subtypes [91]. Additionally, spatial transcriptomic analysis revealed the presence of PLVAP+ cells surrounding adenomyotic lesions. PLVAP expression alters endothelial cells permeability [92], suggesting modified vascular function. All these findings indicate different mechanisms contributing to abnormal vascular dynamics and symptoms such as heavy bleeding, highlighting angiogenesis as a central factor in the pathophysiology of adenomyosis [84,86,90,93]. Notably, Chen et al. [86] detected endothelial tip cells and suggested their maturation is regulated by the activation of the Notch signaling pathway. The Notch pathway’s involvement in vascular alterations has been previously explored [94,95], suggesting it as a potential target for angiogenic therapy in adenomyosis. Furthermore, PLVAP has emerged as a promising target, as its specific inhibition could normalize permeability in uterine endothelial cells affected by adenomyosis [86,92].

Interestingly, Chen et al. identified a distinct subset of SFRP4+IGFBP5^hi^ Natural Killer T (NKT) cells in patients experiencing adenomyosis-related pain, which were absent in patients without such pain. SFRP4 functions as a Wnt-pathway inhibitor that suppresses cell differentiation [96], while *IGFBP5* is a gene associated with neuropathic differentiation [97], suggesting a potential role for these cells in neuroinflammatory processes and nerve fiber proliferation [98]. Single-cell analysis further indicated the potential of these cells to differentiate into neural progenitor cells. Additionally, the genetic heatmap based on pseudotime trajectory analysis revealed consistently high *IGFBP5* expression throughout differentiation along with an increased expression of genes involved in nerve growth and neuroinflammatory pathways at later stages. Among these genes, NEFM was particularly notable due to its association with neuropathic pain [99] and its positive correlation with pain duration in this study. Collectively, these findings propose that SFRP4+IGFBP5^hi^ NKT cells may drive stem cell differentiation toward neurogenic lineages through IGFBP5 expression, thereby contributing to adenomyosis-associated pain [98]. This points to IGFBP5 and NEFM as potential therapeutic targets for managing pain in adenomyosis patients (Figure 2). Furthermore, other studies have suggested that mifepristone is a safe and effective option for improving pain scores in adenomyosis patients compared to placebo [100], while non-steroidal anti-inflammatory drugs have also been reported as effective treatments for dysmenorrhea in this population [101].

Overall, single-cell analyses have revealed the complex interplay of cellular and molecular mechanisms driving adenomyosis, highlighting the potential for therapeutic interventions targeting Wnt signaling, angiogenic pathways, and pain-associated stem cell markers such as *IGFBP5* and *SFRP4*. Despite their promise, sample variability and the incomplete understanding of the biological mechanisms involved have limited the impact of single-cell studies, underscoring the need for more research [84,85,86].

### 3.4. Uterine Fibroids/Leiomyoma

Uterine fibroids (or leiomyomas) are benign smooth muscle tumors and the most common gynecological tumors in reproductive-age women, affecting over 80% of African Americans and ~70% of Caucasian women [102]. Although non-malignant, fibroids significantly impair quality of life by causing symptoms like abnormal uterine bleeding, pelvic pain, and pregnancy complications [103,104]. While research has linked fibroid development to hormonal and genetic factors, the precise molecular mechanisms and cellular contributors remain unclear, limiting diagnostic and therapeutic advances [104,105,106]. ScRNA-seq and spatial transcriptomics have provided transformative insights into fibroid biology, although applications in this area are still emerging.

Recent scRNA-seq-based studies identified critical cellular populations in fibroids, including SMCs, fibroblasts, endothelial cells, immune cells, and progenitor cells [30,107] (Table 1). These studies highlighted the role of SMCs and fibroblasts in ECM deposition, which is a hallmark feature of fibroids [108,109]. SMC subclusters contribute to collagen formation, myofibroblast function, and regulatory processes like proliferation and glycolysis [30]; similarly, fibroblasts exhibit diverse roles in ECM organization, integrin signaling, and axon guidance. The authors observed enhanced cell–cell communication between SMCs and fibroblasts in fibroids with increased signaling pathways related to collagen production, actin remodeling, and semaphorin signaling [110,111]. They also observed amplified immune-fibroblast interactions involving known enhancers (Notch and mTOR pathways) of the pro-fibrotic environment of fibroids [112,113,114]. Furthermore, they identified novel ligand–receptor interactions, such as IGF1-IGF1R, as contributors to disease progression [109]. Dysregulated genes, including *REST*, *CTNNB1*, and *SFRP2*, revealed potential molecular targets for future therapeutic exploration [115,116] (Figure 2).

As hormone-sensitive tumors, estrogen and progesterone play pivotal roles in fibroid development [117,118]. Spatial transcriptomics and scRNA-seq demonstrated the robust expression of estrogen receptor 1 (*ESR1*) and progesterone receptor (*PGR*) in fibroids with significant involvement of the ERK1/ERK2 pathway in mediating hormonal effects and promoting tumor progression. Pseudotime and Gene Ontology (GO) enrichment analyses further indicated that *ESR1* is predominantly expressed in smooth muscle cells (SMCs) and fibroblasts within uterine fibroids, whereas elevated *PGR* expression was detected in fibroid SMCs across all patients but was limited to fibroblasts in only one patient [107]. Conversely, Goad et al. reported an overall reduction in *ESR1* and *PGR* expression in SMCs and fibroblasts within uterine fibroids with the exception of a specific fibroid SMC subcluster that exhibited a higher proportion of *PGR* compared to the myometrium [30]. While the precise association between the differential gene expression and tumor growth remains unclear, these findings suggested paracrine mechanisms through which specific cell populations such as SMC and fibroblasts drive tumor growth, offering insights into the hormonal regulation of fibroids and potential therapeutic targets [30,119,120].

Abnormal angiogenesis, another feature of fibroids, involves abnormal endothelial cell activity also driven by estrogen-activated pathways such as ERK [121,122,123,124]. Notably, ESR2 (ERβ) was predominantly expressed in endothelial cells within fibroids [107], aligning with findings by Valladares et al., who reported high ERβ localization in these cells. Since ERβ has been shown to promote angiogenesis, the ERK1/2 pathway may mediate this process by facilitating endothelial cell migration and proliferation [125]. Immune cells, including T-cells, macrophages, and NK cells, also contribute to the inflammatory and fibrotic microenvironment of fibroids [126,127]. Enhanced interactions between immune cells and fibroblasts further highlight their role in promoting tumor development through signaling pathways such as Notch and mTOR [127].

Fibroids are traditionally considered monoclonal tumors arising from a multipotent progenitor cell that undergoes transformation and differentiation, leading to tumor proliferation [125,128]. In support of this view, Wang et al. identified a progenitor cell population within fibroids, which agreed with earlier reports [129,130]; however, Goad et al. suggested a genetically heterogeneous origin involving MED12 variant-positive progenitor cells that recruit wild-type cells [30,131]. Notably, discrepancies between these studies, particularly regarding *ESR1* and *PGR* expression and the identification of progenitor cell populations, highlight variations in tissue comparisons and sample characteristics. While Wang et al. examined fibroid tissue and its pseudocapsule [107], Goad et al. focused on fibroids and myometrium, analyzing only MED12-mutant fibroids [30]. These methodological differences highlight the need for standardized experimental designs and detailed sample descriptions to ensure reproducibility while emphasizing the complex nature of fibroids and the power of single-cell technologies in uncovering complex cellular and genetic landscapes.

Overall, advanced transcriptomic technologies have significantly enhanced our understanding of fibroid pathogenesis, shedding light on crucial pathways such as ERK1/ERK2, mTOR, and collagen signaling, as well as the roles of specific cell populations in tumor progression. Integration with genome-wide association study data has further enriched our understanding with single-cell and spatial transcriptomics offering valuable insights into fibroid biology and potential avenues for improved patient care [132].

**Table 1:** Summary of single-cell studies of endometriosis, endometrial cancer, adenomyosis and uterine fibroids.

Reference	Samples	Cell Number *	Key Findings
**Endometriosis**			
[43]	EcE (n = 4) and EuE (n = 9) from ovarian EM; peritoneal EM (n = 8) and adjacent regions (n = 6); healthy endometrium (n = 3)	108,497	▪ Identified MUC5B+ epithelial cells as possible progenitor cells of endometriotic lesions ▪ Unique perivascular mural cells in peritoneal lesions promoted angiogenesis and immune cell trafficking, coinciding with increased lymphocyte proportions and upregulated macrophage–regulatory T-cell interactions
[27]	EcE (n = 8) and EuE (n = 10) from ovarian EM and unaffected ovary (n = 4); peritoneal EM (n = 23); healthy peritoneum (n = 4)	373,851	▪ SOX9+/LGR5^+^ epithelial cell subset may participate in EM ▪ Epithelium, stroma, and mesothelial cells of ovarian EM exhibited dysregulated pro-inflammatory pathway activation ▪ *ARID1A* somatic mutations in epithelial cells drive angiogenesis and lymphangiogenesis
[45]	EcE and EuE from ovarian EM (n = 3); healthy endometrium (n = 3)	46,445	▪ Significantly higher proportions of myofibroblasts, pericytes, endothelial cells, and macrophages in EcE compared to EuE and healthy endometrium. ▪ Immunodeficient EM profile
[42]	EcE and EuE from ovarian EM (n = 3); healthy endometrium (n = 3)	55,188	▪ Identified fibroblast subpopulations related to endometriosis development ▪ Diminished immune function and pro-remodeling characteristics of EM
[50]	Menstrual effluent from EM (n = 11); subjects with undiagnosed chronic EM-like symptoms (n = 13); healthy controls (n = 9)	43,054	▪ Reduced decidualized of an IGFBP1+ endometrial stromal cell subset in EM cases suggested impaired decidualization ▪ Endometrial stromal cells in EM exhibited pro-inflammatory and senescent characteristics ▪ Identification of a unique proliferating uNK cell subpopulation in controls, mostly absent in EM
[56]	Peritoneal fluid from EM (n = 1); healthy control (n = 1)	17,530	▪ Identified immune cell dysfunctions in EM, including reduced phagocytosis and cytotoxicity with increased pro-inflammatory and chemotactic responses
[44]	EcE and EuE from ovarian EM (n = 23); healthy endometrium (n = 11)	7030	▪ Increased proportion of ciliated cells in EuE/EcE characterized by diminished expression of estrogen sulfotransferase, likely conferring resistance to apoptosis ▪ Epithelial cells in ectopic lesions stimulated CD4+ T cells, contributing to chronic inflammation
**Endometrial Cancer**			
[68]	EC (n = 4); normal endometrium (n = 2)	41,358	▪ CAF subsets with distinct characteristics maintained frequent communications with malignant cells, facilitating EC progression
[70]	EEC (n = 5); normal endometrium (n = 3)	46,638	▪ SPP1+ TAMs associated with EC tumorigenesis and progression ▪ Robust crosstalk between SPP1+ TAMs and fibroblasts, SMCs, endothelial cells, proliferating T cells, and tumor epithelial cells in the TME
[72]	EC (n = 3)	28,820	▪ NK cells and CD8+ T lymphocytes represented major TIL components in EC patients ▪ Transcriptionally distinct NK cell subsets identified in EC
[65]	EEC (n = 5); matched paratumor samples (n = 3)	30,780	▪ Tumor epithelial cells exhibited the highest CNVs levels ▪ Distinct epithelial cell clusters identified ▪ Enrichment of macrophages and exhausted CD8+ T cells observed in EEC
[133]	EEC (n = 5); USC (n = 1)	150,144	▪ Malignant cells from the same patients revealed variation in chromatin accessibility linked to transcriptional output, highlighting the importance of intratumoral heterogeneity
[66]	EEC (n = 5); atypical endometrial hyperplasia tissues (n = 5); normal endometrium (n = 5)	99,215	▪ Elevated proportion of epithelial cells and increased percentage of CNVs observed in EEC ▪ EEC originated from unciliated glandular epithelial cells and LCN2+/SAA1/2+ cells identified as a subpopulation involved in endometrial tumorigenesis
[26]	EEC-I (n = 7), EEC-II (n = 3), USC (n = 4), and UCCC (n = 3); normal endometrium (n = 1)	146,332	▪ Metabolic, immune, and proliferation pathways enriched in USC, UCCC, and ECC, respectively ▪ Epithelial-to-mesenchymal transition more robustly present in USC ▪ SOD2+ iCAFs exclusive to UCCC and critical for EC malignancy
**Adenomyosis**			
[90]	Adenomyotic EcE and EuE (n = 1); control endometrium (n = 1)	36,781	▪ Cellular cluster with epithelial and endothelial cells in EcE displayed malignant features ▪ Epithelial subcluster enriched in EcE overexpressed motility-related genes ▪ EET and VM found in EcE suggested as novel processes involved in AM pathogenesis
[84]	Adenomyotic EcE and EuE (n = 2); control endometrium (n = 2)	42,260	▪ Endothelial cells involved in pathways related to cancer, epithelial cells in migration functions, and some SMC subclusters associated with vascular SMC contraction ▪ High levels of CNVs in EcE
[98]	Adenomyotic EcE (dysmenorrhea n = 2; no dysmenorrhea n = 2)	27,924	▪ SFRP4+IGFBP5^hi^ NK cells may transform multipotent stem cells into neurogenic cells, leading to AM-related pain
[87]	Adenomyotic EcE and EuE (n = 1)	21,147	▪ Fibrogenesis in AM pathogenesis driven by fibroblasts and FMT alongside the contribution of SMC and immune cells
[85]	Adenomyotic EcE, EuE, and MM (n = 3)	66,000	▪ Highly heterogeneous fibroblasts represented the main component of the AM endometrium ▪ Wnt pathway proposed as a critical regulator of AM pathophysiology
[86]	Adenomyotic EcE, EuE, MM, and EMJ (n = 3); control EuE, MM, and EMJ (n = 1)	54,658	▪ Identification of unique LGR5^+^ epithelial cells, PKIB^+^ invasive stromal cells, and WFDC1^+^ progenitor cells within EcE ▪ Abnormal angiogenic function and altered cell–cell communications in adenomyotic tissue
**Uterine Fibroids**			
[30]	MED12^+^ UF (n = 5); adjacent MM (n = 5)	39,209	▪ Description of lymphatic endothelial cells within UF, previously reported only in MM ▪ Fibroblasts drove UF microenvironmental alterations via interactions with SMCs, endothelial cells, and immune cells ▪ UF genetic heterogeneity suggested a non-monoclonal origin for these tumors
[107]	UF (n = 2), pseudocapsule (n = 2), and MM (n = 1)	26,371	▪ ERK1/ERK2 pathway may mediate UF development and angiogenesis via ER and PGR signaling ▪ IGF1 and IGF1R represented the most significant ligand–receptor pair identified in fibroblast interactions
[132]	MED12^+^ UF (n = 5); MM (n = 5)	48,258	▪ SMCs contained the most highly expressed GWAS target genes in UF samples ▪ Cell type-specific modules of enriched GWAS candidate genes identified

* Number of cells analyzed after quality control filters. Abbreviations: AM: adenomyosis; CAFs: cancer-associated fibroblasts; CNVs: copy number variations; EC: endometrial cancer; EcE: ectopic endometrium; EEC: endometrioid endometrial cancer; EEC-I: well-differentiated EEC; EEC-II: poorly differentiated ECC; EET: epithelial–endothelial transition; EM: endometriosis; EMJ: endometrial–myometrial junction; EuE: eutopic endometrium; FMT: fibroblast-to-myofibroblast transition; GWAS: genome-wide association study; MM: myometrium; NK: natural killer cells; PGR: progesterone receptor; SMCs: smooth muscle cells; TAMs: tumor-associated macrophages; TILs: tumor-infiltrating lymphocytes; TME: tumor microenvironment; UCCC: uterine clear cell carcinoma; UF: uterine fibroids; uNK: uterine natural killer cells; USC: uterine serous carcinoma; VM: vasculogenic mimicry.

## 4. Challenges and Constraints in Current Single-Cell Technologies

While scRNA-seq has transformed biological research by supporting the high-resolution analysis of individual cells [18], constraints remain, including the presence of biological noise (e.g., gene expression variability and cell state differences) and technical limitations (e.g., partial RNA capture and amplification bias) [134]. Said issues can lead to the generation of incomplete datasets and the underrepresentation of specific RNA species; moreover, sample handling, procurement, and processing delays can reduce RNA quality, further affecting the reliability of the results [135].

Batch effects arising from variations in sample processing conditions and sequencing protocols pose significant challenges to scRNA-seq analysis, confounding biological signals and requiring careful design and experimental replication to mitigate any impact [134]. Biological factors (e.g., cell cycle effects) further complicate analyses by obscuring real differences between cells. Technical limitations (e.g., the inability to capture certain RNA species) contribute to data sparsity with observed zeros potentially representing biological absence or technical dropouts [136]. Beyond these inherent biological and technical constraints, experimental design also plays a critical role in influencing the comparability and interpretation of findings across studies. For example, in single-cell studies of uterine fibroids, comparisons of gene expression between fibroids and either healthy myometrium [30] or surrounding pseudocapsules [107] produced conflicting results regarding *ESR1* and *PGR* expression. Similarly, studies on endometrial cancer reported inconsistent findings about CD8+ T cells with some indicating reduced proportions [66] and others suggesting an enrichment of these cells among tumor-infiltrating lymphocytes [72]. These discrepancies are partly due to the variations in the control samples used, highlighting how differences in sample selection can impact outcomes and reproducibility. Furthermore, the diversity of scRNA-seq platforms—each with unique strengths, limitations, and requirements—further complicates data integration/standardization [134,135]. Addressing these challenges through normalization, correction, and informed platform selection remains critical to generating reliable and interpretable results [137].

## 5. Future Perspectives: Addressing Research Gaps for Clinical Implementation

Over recent decades, advancements in omics-based technologies have significantly enhanced our understanding of the molecular mechanisms underlying various physiological and pathological processes. In particular, single-cell technologies have shown remarkable potential in studying complex disorders with applications spanning therapeutic target identification, drug screening, clinical model development, and diagnostics [138]. These advancements have shown particular promise in gynecology and reproductive medicine, where scRNA-seq and multi-omics-based approaches have offered high-resolution insight into disease pathogenesis. Notably, single-cell technologies have enabled the characterization of tumor heterogeneity, the identification of disease-specific cell and molecular subtypes, and an understanding of immune microenvironments and therapy resistance evolution in gynecological cancers such as ovarian, endometrial, and cervical cancers [138,139,140,141,142,143]. These insights have particular value when addressing challenges related to metastasis and drug resistance. Multi-omics single-cell technologies, which can integrate genomic, transcriptomic, proteomic, epigenomic and metabolomic data from the same single cell, offer a systems biology approach to deciphering the intricate mechanisms underlying gynecological disorders [144]. Additionally, scRNA-seq profiling of endometrial organoids has allowed researchers to benchmark in vitro responses against in vivo data, enhancing the relevance of these models for the study of endometrial function [34]. These advances highlight the potential of single-cell approaches to refine preclinical models and improve translational research outcomes.

Nonetheless, the analysis and interpretation of vast large-scale omics data concerning uterine diseases have been complicated by the cyclic regulation of hormones in addition to multiple other factors, such as genetic variability [145], environmental factors [146], and microbiome composition [147], among others. This challenge is further compounded by individual genetic differences, which contribute to diverse biological responses. In this context, Sengupta et al. recently proposed a tool that examines the interconnected roles of hormonal regulation, environmental factors, genetic predisposition (including DNA composition and epigenetic changes), health implications, and the resulting biological effects [144]. Moreover, wider accessibility to public repositories, such as Gene Expression Omnibus, ArrayExpress, and single-cell-focused resources like Tabula Muris, Non-Human Primate SC Atlas, Pan-Cancer Blueprint, and the Human Tumor Atlas Network, offers significant opportunities to overcome data accessibility challenges and leverage existing data for clinical applications [138].

Looking forward, the integration of single-cell technologies into clinical workflows is expected to drive significant advancements in precision medicine. These tools will enable the identification of novel therapeutic targets, including the emerging proteolysis targeting chimera (PROTAC) probe technology [148], which has been utilized to successfully degrade a variety of pathogenic proteins. Furthermore, single-cell technologies can aid in the characterization of heterogeneously manifested human traits and the refinement of cell differentiation trajectories. By elucidating the genetic drivers of metastasis, treatment resistance, and tumor progression in gynecological cancers, single-cell technologies will pave the way for personalized treatments with improved efficacy and reduced side effects. The continued development of sophisticated computational tools, standardized workflows, and interdisciplinary collaborations will be crucial for translating these innovations into routine clinical practice, heralding a new era of personalized medicine.

## 6. Conclusions

Gynecological disorders such as endometriosis, ovarian cancer, and uterine fibroids possess complex and multicellular etiologies, making characterization a challenging prospect. Single-cell technologies have provided a powerful approach to address this complexity by providing detailed insights into the cellular and molecular diversity within gynecological tissues. Combined with advanced multiplexed approaches, single-cell protocols enable the high-resolution analysis of various cell populations, uncovering essential cell types and signaling pathways driving disease mechanisms.

A key aspect of these disorders is the significant overlap in underlying mechanisms, including excessive collagen deposition leading to fibrosis, heightened inflammatory responses, and immune dysregulation. These shared features suggest common biomarkers and therapeutic targets that could address these conditions collectively. For instance, targeting the TGF-β signaling pathway or genes associated with extracellular matrix (ECM) remodeling, such as fibronectin, collagen, and α smooth muscle actin, holds promise for mitigating fibrosis [149]. Fibrosis represents a key pathological hallmark, impacting tissue architecture and function, ultimately influencing fertility and quality of life [150,151]. Immune dysfunction and chronic inflammation further exacerbate disease progression, impairing immune cell function and disrupting tissue homeostasis [152]. Blocking drivers of chronic inflammation, such as the NLRP3 inflammasome [153] or the NF-κB signaling pathway [154], represents a promising therapeutic strategy. However, while several NLRP3 inhibitors have been developed, their clinical use has been constrained by toxicity and safety concerns [153]. Similarly, targeting the NF-κB pathway poses challenges due to its involvement in critical biological processes, risking off-target effects [155]. Based on this premise, future efforts must focus on designing structure-based direct inhibitors with improved specificity and minimized side effects to optimize therapeutic efficacy. Additionally, profiling preclinical models at the single-cell level will help to identify those most accurately mirroring human gynecological disease biology.

This cellular-level analysis of human samples supports the development of personalized medicine by revealing novel biomarkers and aiding patient stratification based on prognosis/expected response to treatment. By dissecting disease complexity at the cellular level, single-cell technologies aid the discovery of novel biomarkers and help to clarify the fundamental biology of gynecological conditions, paving the way for targeted therapeutic interventions. Consequently, single-cell technologies will remain pivotal in identifying biological targets and carrying out their functional characterization.

Emerging evidence highlights the connection between these disorders and their impact on fertility [156]. Given the associations between these conditions, fertility preservation, and overall health, further research is required to elucidate these mechanisms and optimize therapeutic approaches.

## Data Availability

No new data were created or analyzed in this study.

## References

[1] Habiba M., Heyn R., Bianchi P., Brosens I., Benagiano G. (2021). The Development of the Human Uterus: Morphogenesis to Menarche. Hum. Reprod. Update.

[2] Robboy S.J., Kurita T., Baskin L., Cunha G.R. (2017). New Insights into Human Female Reproductive Tract Development. Differentiation.

[3] Yamaguchi M., Yoshihara K., Suda K., Nakaoka H., Yachida N., Ueda H., Sugino K., Mori Y., Yamawaki K., Tamura R. (2021). Three-Dimensional Understanding of the Morphological Complexity of the Human Uterine Endometrium. iScience.

[4] Ruiz-Alonso M., Blesa D., Simón C. (2012). The Genomics of the Human Endometrium. Biochim. Biophys. Acta.

[5] Renthal N.E., Chen C.C., Williams K.C., Gerard R.D., Prange-Kiel J., Mendelson C.R. (2010). MiR-200 Family and Targets, ZEB1 and ZEB2, Modulate Uterine Quiescence and Contractility during Pregnancy and Labor. Proc. Natl. Acad. Sci. USA.

[6] Maiti K., Paul J.W., Read M., Chan E.C., Riley S.C., Nahar P., Smith R. (2011). G-1-Activated Membrane Estrogen Receptors Mediate Increased Contractility of the Human Myometrium. Endocrinology.

[7] Talati C., Carvalho J.C.A., Luca A., Balki M. (2019). The Effect of Intermittent Oxytocin Pretreatment on Oxytocin-Induced Contractility of Human Myometrium In Vitro. Anesth. Analg..

[8] Hrdlickova R., Toloue M., Tian B. (2017). RNA-Seq Methods for Transcriptome Analysis. Wiley Interdiscip. Rev. RNA.

[9] Evans G.E., Phillipson G.T.M., Sykes P.H., McNoe L.A., Print C.G., Evans J.J. (2018). Does the Endometrial Gene Expression of Fertile Women Vary within and between Cycles?. Hum. Reprod..

[10] Li F., Gao W., Li Y., Wang Y., Liu L., Zhang X. (2023). Potential Biomarkers and Endometrial Immune Microenvironment in Recurrent Implantation Failure. Biomolecules.

[11] Herndon C.N., Aghajanova L., Balayan S., Erikson D., Barragan F., Goldfien G., Vo K.C., Hawkins S., Giudice L.C. (2016). Global Transcriptome Abnormalities of the Eutopic Endometrium from Women with Adenomyosis. Reprod. Sci..

[12] Nookaew I., Papini M., Pornputtapong N., Scalcinati G., Fagerberg L., Uhlén M., Nielsen J. (2012). A Comprehensive Comparison of RNA-Seq-Based Transcriptome Analysis from Reads to Differential Gene Expression and Cross-Comparison with Microarrays: A Case Study in Saccharomyces Cerevisiae. Nucleic Acids Res..

[13] Sanchez-Ribas I., Diaz-Gimeno P., Sebastián-León P., Mercader A., Quiñonero A., Ballesteros A., Pellicer A., Domínguez F. (2019). Transcriptomic Behavior of Genes Associated with Chromosome 21 Aneuploidies in Early Embryo Development. Fertil. Steril..

[14] Suhorutshenko M., Kukushkina V., Velthut-Meikas A., Altmäe S., Peters M., Mägi R., Krjutškov K., Koel M., Codoñer F.M., Martinez-Blanch J.F. (2018). Endometrial Receptivity Revisited: Endometrial Transcriptome Adjusted for Tissue Cellular Heterogeneity. Hum. Reprod..

[15] Choi M.R., Chang H.J., Heo J.H., Yum S.H., Jo E., Kim M., Lee S.R. (2024). Expression Profiling of Coding and Noncoding RNAs in the Endometrium of Patients with Endometriosis. Int. J. Mol. Sci..

[16] Xiang Y., Sun Y., Yang B., Yang Y., Zhang Y., Yu T., Huang H., Zhang J., Xu H. (2019). Transcriptome Sequencing of Adenomyosis Eutopic Endometrium: A New Insight into Its Pathophysiology. J. Cell. Mol. Med..

[17] Machado-Lopez A., Alonso R., Lago V., Jimenez-Almazan J., Garcia M., Monleon J., Lopez S., Barcelo F., Torroba A., Ortiz S. (2022). Integrative Genomic and Transcriptomic Profiling Reveals a Differential Molecular Signature in Uterine Leiomyoma versus Leiomyosarcoma. Int. J. Mol. Sci..

[18] Shapiro E., Biezuner T., Linnarsson S. (2013). Single-Cell Sequencing-Based Technologies Will Revolutionize Whole-Organism Science. Nat. Rev. Genet..

[19] Kulkarni A., Anderson A.G., Merullo D.P., Konopka G. (2019). Beyond Bulk: A Review of Single Cell Transcriptomics Methodologies and Applications. Curr. Opin. Biotechnol..

[20] Truong D.D., Lamhamedi-Cherradi S.E., Porter R.W., Krishnan S., Swaminathan J., Gibson A., Lazar A.J., Livingston J.A., Gopalakrishnan V., Gordon N. (2023). Dissociation Protocols Used for Sarcoma Tissues Bias the Transcriptome Observed in Single-Cell and Single-Nucleus RNA Sequencing. BMC Cancer.

[21] Grindberg R.V., Yee-Greenbaum J.L., McConnell M.J., Novotny M., O’Shaughnessy A.L., Lambert G.M., Araúzo-Bravo M.J., Lee J., Fishman M., Robbins G.E. (2013). RNA-Sequencing from Single Nuclei. Proc. Natl. Acad. Sci. USA.

[22] Lake B.B., Codeluppi S., Yung Y.C., Gao D., Chun J., Kharchenko P.V., Linnarsson S., Zhang K. (2017). A Comparative Strategy for Single-Nucleus and Single-Cell Transcriptomes Confirms Accuracy in Predicted Cell-Type Expression from Nuclear RNA. Sci. Rep..

[23] Su M., Pan T., Chen Q.Z., Zhou W.W., Gong Y., Xu G., Yan H.Y., Li S., Shi Q.Z., Zhang Y. (2022). Data Analysis Guidelines for Single-Cell RNA-Seq in Biomedical Studies and Clinical Applications. Mil. Med. Res..

[24] Tang F., Barbacioru C., Wang Y., Nordman E., Lee C., Xu N., Wang X., Bodeau J., Tuch B.B., Siddiqui A. (2009). MRNA-Seq Whole-Transcriptome Analysis of a Single Cell. Nat. Methods.

[25] Punzon-Jimenez P., Machado-Lopez A., Perez-Moraga R., Llera-Oyola J., Grases D., Galvez-Viedma M., Sibai M., Satorres-Perez E., Lopez-Agullo S., Badenes R. (2024). Effect of Aging on the Human Myometrium at Single-Cell Resolution. Nat. Commun..

[26] Ren F., Wang L., Wang Y., Wang J., Wang Y., Song X., Zhang G., Nie F., Lin S. (2024). Single-Cell Transcriptome Profiles the Heterogeneity of Tumor Cells and Microenvironments for Different Pathological Endometrial Cancer and Identifies Specific Sensitive Drugs. Cell Death Dis..

[27] Fonseca M.A.S., Haro M., Wright K.N., Lin X., Abbasi F., Sun J., Hernandez L., Orr N.L., Hong J., Choi-Kuaea Y. (2023). Single-Cell Transcriptomic Analysis of Endometriosis. Nat. Genet..

[28] Jones R.C., Karkanias J., Krasnow M.A., Pisco A.O., Quake S.R., Salzman J., Yosef N., Bulthaup B., Brown P., Harper W. (2022). The Tabula Sapiens: A Multiple-Organ, Single-Cell Transcriptomic Atlas of Humans. Science.

[29] Pique-Regi R., Romero R., Garcia-Flores V., Peyvandipour A., Tarca A.L., Pusod E., Galaz J., Miller D., Bhatti G., Para R. (2022). A Single-Cell Atlas of the Myometrium in Human Parturition. JCI Insight.

[30] Goad J., Rudolph J., Zandigohar M., Tae M., Dai Y., Wei J.J., Bulun S.E., Chakravarti D., Rajkovic A. (2022). Single-Cell Sequencing Reveals Novel Cellular Heterogeneity in Uterine Leiomyomas. Hum. Reprod..

[31] Karlsson M., Zhang C., Méar L., Zhong W., Digre A., Katona B., Sjöstedt E., Butler L., Odeberg J., Dusart P. (2021). A Single-Cell Type Transcriptomics Map of Human Tissues. Sci. Adv..

[32] Krjutškov K., Katayama S., Saare M., Vera-Rodriguez M., Lubenets D., Samuel K., Laisk-Podar T., Teder H., Einarsdottir E., Salumets A. (2016). Single-Cell Transcriptome Analysis of Endometrial Tissue. Hum. Reprod..

[33] Wang W., Vilella F., Alama P., Moreno I., Mignardi M., Isakova A., Pan W., Simon C., Quake S.R. (2020). Single-Cell Transcriptomic Atlas of the Human Endometrium during the Menstrual Cycle. Nat. Med..

[34] Garcia-Alonso L., Handfield L.-F., Roberts K., Nikolakopoulou K., Fernando R.C., Gardner L., Woodhams B., Arutyunyan A., Polanski K., Hoo R. (2021). Mapping the Temporal and Spatial Dynamics of the Human Endometrium in Vivo and in Vitro. Nat. Genet..

[35] Marečková M., Garcia-Alonso L., Moullet M., Lorenzi V., Petryszak R., Sancho-Serra C., Oszlanczi A., Icoresi Mazzeo C., Wong F.C.K., Kelava I. (2024). An Integrated Single-Cell Reference Atlas of the Human Endometrium. Nat. Genet..

[36] Ji K., Zhong J., Cui L., Wang X., Chen L.N., Wen B., Yang F., Deng W., Pan X., Wang L. (2024). Exploring Myometrial Microenvironment Changes at the Single-Cell Level from Nonpregnant to Term Pregnant States. Physiol. Genom..

[37] Ulrich N.D., Vargo A., Ma Q., Shen Y., Hannum D.F., Gurczynski S.J., Moore B.B., Schon S., Lieberman R., Shikanov A. (2024). Cellular Heterogeneity and Dynamics of the Human Uterus in Healthy Premenopausal Women. bioRxiv.

[38] World Health Organization Endometriosis. https://www.who.int/news-room/fact-sheets/detail/endometriosis.

[39] Barnard M.E., Farland L.V., Yan B., Wang J., Trabert B., Doherty J.A., Meeks H.D., Madsen M., Guinto E., Collin L.J. (2024). Endometriosis Typology and Ovarian Cancer Risk. JAMA.

[40] Linder A., Westbom-Fremer S., Mateoiu C., Olsson Widjaja A., Österlund T., Veerla S., Ståhlberg A., Ulfenborg B., Hedenfalk I., Sundfeldt K. (2024). Genomic Alterations in Ovarian Endometriosis and Subsequently Diagnosed Ovarian Carcinoma. Hum. Reprod..

[41] Zondervan K.T., Becker C.M., Missmer S.A. (2020). Endometriosis. N. Engl. J. Med..

[42] Ma J., Zhang L., Zhan H., Mo Y., Ren Z., Shao A., Lin J. (2021). Single-Cell Transcriptomic Analysis of Endometriosis Provides Insights into Fibroblast Fates and Immune Cell Heterogeneity. Cell Biosci..

[43] Tan Y., Flynn W.F., Sivajothi S., Luo D., Bozal S.B., Davé M., Luciano A.A., Robson P., Luciano D.E., Courtois E.T. (2022). Single-Cell Analysis of Endometriosis Reveals a Coordinated Transcriptional Programme Driving Immunotolerance and Angiogenesis across Eutopic and Ectopic Tissues. Nat. Cell Biol..

[44] Yan J., Zhou L., Liu M., Zhu H., Zhang X., Cai E., Xu X., Chen T., Cheng H., Liu J. (2024). Single-Cell Analysis Reveals Insights into Epithelial Abnormalities in Ovarian Endometriosis. Cell Rep..

[45] Zhu S., Wang A., Xu W., Hu L., Sun J., Wang X. (2023). The Heterogeneity of Fibrosis and Angiogenesis in Endometriosis Revealed by Single-Cell RNA-Sequencing. Biochim. Biophys..

[46] Zhang Z., Suo L., Chen Y., Zhu L., Wan G., Han X. (2019). Endometriotic Peritoneal Fluid Promotes Myofibroblast Differentiation of Endometrial Mesenchymal Stem Cells. Stem Cells Int..

[47] Zhang Z., Wang J., Chen Y., Suo L., Chen H., Zhu L., Wan G., Han X. (2019). Activin a Promotes Myofibroblast Differentiation of Endometrial Mesenchymal Stem Cells via STAT3-Dependent Smad/CTGF Pathway. Cell Commun. Signal..

[48] Klemmt P.A.B., Carver J.G., Kennedy S.H., Koninckx P.R., Mardon H.J. (2006). Stromal Cells from Endometriotic Lesions and Endometrium from Women with Endometriosis Have Reduced Decidualization Capacity. Fertil. Steril..

[49] Warren L.A., Shih A., Renteira S.M., Seckin T., Blau B., Simpfendorfer K., Lee A., Metz C.N., Gregersen P.K. (2018). Analysis of Menstrual Effluent: Diagnostic Potential for Endometriosis. J. Mol. Med..

[50] Shih A.J., Adelson R.P., Vashistha H., Khalili H., Nayyar A., Puran R., Herrera R., Chatterjee P.K., Lee A.T., Truskinovsky A.M. (2022). Single-Cell Analysis of Menstrual Endometrial Tissues Defines Phenotypes Associated with Endometriosis. BMC Med..

[51] Evans J., Salamonsen L.A., Winship A., Menkhorst E., Nie G., Gargett C.E., Dimitriadis E. (2016). Fertile Ground: Human Endometrial Programming and Lessons in Health and Disease. Nat. Rev. Endocrinol..

[52] Steinbuch S.C., Lüß A.M., Eltrop S., Götte M., Kiesel L. (2024). Endometriosis-Associated Ovarian Cancer: From Molecular Pathologies to Clinical Relevance. Int. J. Mol. Sci..

[53] Suryawanshi S., Huang X., Elishaev E., Budiu R.A., Zhang L., Kim S.H., Donnellan N., Mantia-Smaldone G., Ma T., Tseng G. (2014). Complement Pathway Is Frequently Altered in Endometriosis and Endometriosis-Associated Ovarian Cancer. Clin. Cancer Res..

[54] Saavalainen L., Lassus H., But A., Tiitinen A., Härkki P., Gissler M., Pukkala E., Heikinheimo O. (2018). Risk of Gynecologic Cancer According to the Type of Endometriosis. Obstet. Gynecol..

[55] Abramiuk M., Grywalska E., Małkowska P., Sierawska O., Hrynkiewicz R., Niedźwiedzka-Rystwej P. (2022). The Role of the Immune System in the Development of Endometriosis. Cells.

[56] Zou G., Wang J., Xu X., Xu P., Zhu L., Yu Q., Peng Y., Guo X., Li T., Zhang X. (2021). Cell Subtypes and Immune Dysfunction in Peritoneal Fluid of Endometriosis Revealed by Single-Cell RNA-Sequencing. Cell Biosci..

[57] Sung H., Ferlay J., Siegel R.L., Laversanne M., Soerjomataram I., Jemal A., Bray F. (2021). Global Cancer Statistics 2020: GLOBOCAN Estimates of Incidence and Mortality Worldwide for 36 Cancers in 185 Countries. CA Cancer J. Clin..

[58] World Cancer Research Fund International Endometrial Cancer Statistics. https://www.wcrf.org/preventing-cancer/cancer-statistics/endometrial-cancer-statistics/.

[59] Mazidimoradi A., Momenimovahed Z., Khalajinia Z., Allahqoli L., Salehiniya H., Alkatout I. (2024). The Global Incidence, Mortality, and Burden of Uterine Cancer in 2019 and Correlation with SDI, Tobacco, Dietary Risks, and Metabolic Risk Factors: An Ecological Study. Health Sci. Rep..

[60] Ye J., Peng H., Huang X., Qi X. (2022). The Association between Endometriosis and Risk of Endometrial Cancer and Breast Cancer: A Meta-Analysis. BMC Womens Health.

[61] Bianco B., Barbosa C.P., Trevisan C.M., Laganà A.S., Montagna E. (2020). Endometrial Cancer: A Genetic Point of View. Transl. Cancer Res..

[62] Cao S., Fan Y., Zhang Y., Ruan J., Mu Y., Li J. (2023). Recurrence and Survival of Patients with Stage III Endometrial Cancer after Radical Surgery Followed by Adjuvant Chemo- or Chemoradiotherapy: A Systematic Review and Meta-Analysis. BMC Cancer.

[63] Bogani G., Ray-Coquard I., Concin N., Ngoi N.Y.L., Morice P., Enomoto T., Takehara K., Denys H., Lorusso D., Coleman R. (2022). Clear Cell Carcinoma of the Endometrium. Gynecol. Oncol..

[64] Jamaluddin M.F.B., Ko Y.A., Ghosh A., Syed S.M., Ius Y., O’Sullivan R., Netherton J.K., Baker M.A., Nahar P., Jaaback K. (2022). Proteomic and Functional Characterization of Intra-Tumor Heterogeneity in Human Endometrial Cancer. Cell Rep. Med..

[65] Guo Y.E., Li Y., Cai B., He Q., Chen G., Wang M., Wang K., Wan X., Yan Q. (2021). Phenotyping of Immune and Endometrial Epithelial Cells in Endometrial Carcinomas Revealed by Single-Cell RNA Sequencing. Aging.

[66] Ren X., Liang J., Zhang Y., Jiang N., Xu Y., Qiu M., Wang Y., Zhao B., Chen X. (2022). Single-Cell Transcriptomic Analysis Highlights Origin and Pathological Process of Human Endometrioid Endometrial Carcinoma. Nat. Commun..

[67] Mittal V. (2018). Epithelial Mesenchymal Transition in Tumor Metastasis. Annu. Rev. Pathol..

[68] Yu Z., Zhang J., Zhang Q., Wei S., Shi R., Zhao R., An L., Grose R., Feng D., Wang H. (2022). Single-Cell Sequencing Reveals the Heterogeneity and Intratumoral Crosstalk in Human Endometrial Cancer. Cell Prolif..

[69] Dey D.K., Krause D., Rai R., Choudhary S., Dockery L.E., Chandra V. (2023). The Role and Participation of Immune Cells in the Endometrial Tumor Microenvironment. Pharmacol. Ther..

[70] Wu Q., Jiang G., Sun Y., Li B. (2023). Reanalysis of Single-Cell Data Reveals Macrophage Subsets Associated with the Immunotherapy Response and Prognosis of Patients with Endometrial Cancer. Exp. Cell Res..

[71] Sun T., Sun B.C., Zhao X.L., Zhao N., Dong X.Y., Che N., Yao Z., Ma Y.M., Gu Q., Zong W.K. (2011). Promotion of Tumor Cell Metastasis and Vasculogenic Mimicry by Way of Transcription Coactivation by Bcl-2 and Twist1: A Study of Hepatocellular Carcinoma. Hepatology.

[72] Jiang F., Jiao Y., Yang K., Mao M., Yu M., Cao D., Xiang Y. (2022). Single-Cell Profiling of the Immune Atlas of Tumor-Infiltrating Lymphocytes in Endometrial Carcinoma. Cancers.

[73] Horeweg N., Workel H.H., Loiero D., Church D.N., Vermij L., Léon-Castillo A., Krog R.T., de Boer S.M., Nout R.A., Powell M.E. (2022). Tertiary Lymphoid Structures Critical for Prognosis in Endometrial Cancer Patients. Nat. Commun..

[74] Vercellini P., Viganò P., Somigliana E., Daguati R., Abbiati A., Fedele L. (2006). Adenomyosis: Epidemiological Factors. Best. Pract. Res. Clin. Obstet. Gynaecol..

[75] Mehasseb M.K., Bell S.C., Pringle J.H., Habiba M.A. (2010). Uterine Adenomyosis Is Associated with Ultrastructural Features of Altered Contractility in the Inner Myometrium. Fertil. Steril..

[76] Struble J., Reid S., Bedaiwy M.A. (2016). Adenomyosis: A Clinical Review of a Challenging Gynecologic Condition. J. Minim. Invasive Gynecol..

[77] Kok V.C., Tsai H.J., Su C.F., Lee C.K. (2015). The Risks for Ovarian, Endometrial, Breast, Colorectal, and Other Cancers in Women with Newly Diagnosed Endometriosis or Adenomyosis: A Population-Based Study. Int. J. Gynecol. Cancer.

[78] Hermens M., van Altena A.M., Bulten J., van Vliet H.A.A.M., Siebers A.G., Bekkers R.L.M. (2021). Increased Incidence of Ovarian Cancer in Both Endometriosis and Adenomyosis. Gynecol. Oncol..

[79] Yu O., Schulze-Rath R., Grafton J., Hansen K., Scholes D., Reed S.D. (2020). Adenomyosis Incidence, Prevalence and Treatment: United States Population-Based Study 2006–2015. Am. J. Obstet. Gynecol..

[80] Ferenczy A. (1998). Pathophysiology of Adenomyosis. Hum. Reprod. Update.

[81] García-Solares J., Donnez J., Donnez O., Dolmans M.M. (2018). Pathogenesis of Uterine Adenomyosis: Invagination or Metaplasia?. Fertil. Steril..

[82] Maclean A., Barzilova V., Patel S., Bates F., Hapangama D.K. (2023). Characterising the Immune Cell Phenotype of Ectopic Adenomyosis Lesions Compared with Eutopic Endometrium: A Systematic Review. J. Reprod. Immunol..

[83] Bulun S.E., Yildiz S., Adli M., Wei J.J. (2021). Adenomyosis Pathogenesis: Insights from next-Generation Sequencing. Hum. Reprod. Update.

[84] Lin J., Liu L., Zheng F., Chen S., Yang W., Li J., Mo S., Zeng D.Y. (2022). Exploration the Global Single-Cell Ecological Landscape of Adenomyosis-Related Cell Clusters by Single-Cell RNA Sequencing. Front. Genet..

[85] Yildiz S., Kinali M., Wei J.J., Milad M., Yin P., Adli M., Bulun S.E. (2023). Adenomyosis: Single-Cell Transcriptomic Analysis Reveals a Paracrine Mesenchymal-Epithelial Interaction Involving the WNT/SFRP Pathway. Fertil. Steril..

[86] Chen T., Xu Y., Xu X., Wang J., Qiu Z., Yu Y., Jiang X., Shao W., Bai D., Wang M. (2024). Comprehensive Transcriptional Atlas of Human Adenomyosis Deciphered by the Integration of Single-Cell RNA-Sequencing and Spatial Transcriptomics. Protein Cell.

[87] Niu W., Zhang Y., Liu H., Liang N., Xu L., Li Y., Yao W., Shi W., Liu Z. (2023). Single-Cell Profiling Uncovers the Roles of Endometrial Fibrosis and Microenvironmental Changes in Adenomyosis. J. Inflamm. Res..

[88] Oh S.J., Shin J.H., Kim T.H., Lee H.S., Yoo J.Y., Ahn J.Y., Broaddus R.R., Taketo M.M., Lydon J.P., Leach R.E. (2013). β -Catenin Activation Contributes to the Pathogenesis of Adenomyosis through Epithelial-Mesenchymal Transition. J. Pathol..

[89] Zhang W.J., Chen S.J., Zhou S.C., Wu S.Z., Wang H. (2021). Inflammasomes and Fibrosis. Front. Immunol..

[90] Liu Z., Sun Z., Liu H., Niu W., Wang X., Liang N., Wang X., Wang Y., Shi Y., Xu L. (2021). Single-Cell Transcriptomic Analysis of Eutopic Endometrium and Ectopic Lesions of Adenomyosis. Cell Biosci..

[91] Lee H.W., Xu Y., He L., Choi W., Gonzalez D., Jin S.W., Simons M. (2021). Role of Venous Endothelial Cells in Developmental and Pathologic Angiogenesis. Circulation.

[92] Denzer L., Muranyi W., Schroten H., Schwerk C. (2023). The Role of PLVAP in Endothelial Cells. Cell Tissue Res..

[93] Huang T.S., Chen Y.J., Chou T.Y., Chen C.Y., Li H.Y., Huang B.S., Tsai H.W., Lan H.Y., Chang C.H., Twu N.F. (2014). Oestrogen-Induced Angiogenesis Promotes Adenomyosis by Activating the Slug-VEGF Axis in Endometrial Epithelial Cells. J. Cell. Mol. Med..

[94] Noguera-Troise I., Daly C., Papadopoulos N.J., Coetzee S., Boland P., Gale N.W., Chieh Lin H., Yancopoulos G.D., Thurston G. (2006). Blockade of Dll4 Inhibits Tumour Growth by Promoting Non-Productive Angiogenesis. Nature.

[95] Li J.L., Sainson R.C.A., Shi W., Leek R., Harrington L.S., Preusser M., Biswas S., Turley H., Heikamp E., Hainfellner J.A. (2007). Delta-like 4 Notch Ligand Regulates Tumor Angiogenesis, Improves Tumor Vascular Function, and Promotes Tumor Growth in Vivo. Cancer Res..

[96] Sundaram S.M., Varier L., Fathima K.Z., Dharmarajan A., Warrier S. (2023). Short Peptide Domains of the Wnt Inhibitor SFRP4 Target Ovarian Cancer Stem Cells by Neutralizing the Wnt β-Catenin Pathway, Disrupting the Interaction between β-Catenin and CD24 and Suppressing Autophagy. Life Sci..

[97] Li J., Diao S., Yang H., Cao Y., Du J., Yang D. (2019). IGFBP5 Promotes Angiogenic and Neurogenic Differentiation Potential of Dental Pulp Stem Cells. Dev. Growth Differ..

[98] Chen Y., Zhu J., Chen L., Shen Y., Zhang J., Wang Q. (2022). SFRP4+IGFBP5^hi^ NKT Cells Induced Neural-like Cell Differentiation to Contribute to Adenomyosis Pain. Front. Immunol..

[99] Cui C.Y., Liu X., Peng M.H., Liu Q., Zhang Y. (2022). Identification of Key Candidate Genes and Biological Pathways in Neuropathic Pain. Comput. Biol. Med..

[100] Che X., Wang J., Sun W., He J., Wang Q., Zhu D., Zhu W., Zhang J., Dong J., Xu J. (2023). Effect of Mifepristone vs Placebo for Treatment of Adenomyosis with Pain Symptoms: A Randomized Clinical Trial. JAMA Netw. Open.

[101] Moldassarina R.S. (2023). Modern View on the Diagnostics and Treatment of Adenomyosis. Arch. Gynecol. Obstet..

[102] Baird D.D., Dunson D.B., Hill M.C., Cousins D., Schectman J.M. (2003). High Cumulative Incidence of Uterine Leiomyoma in Black and White Women: Ultrasound Evidence. Am. J. Obstet. Gynecol..

[103] Benson C.B., Chow J.S., Chang-Lee W., Iii J.A.H., Doubilet P.M. (2001). Outcome of Pregnancies in Women with Uterine Leiomyomas Identified by Sonography in the First Trimester. J. Clin. Ultrasound.

[104] Marsh E.E., Bulun S.E. (2006). Steroid Hormones and Leiomyomas. Obstet. Gynecol. Clin. N. Am..

[105] Parikh T.P., Malik M., Britten J., Aly J.M., Pilgrim J., Catherino W.H. (2020). Steroid Hormones and Hormone Antagonists Regulate the Neural Marker Neurotrimin in Uterine Leiomyoma. Fertil. Steril..

[106] Machado-Lopez A., Simón C., Mas A. (2021). Molecular and Cellular Insights into the Development of Uterine Fibroids. Int. J. Mol. Sci..

[107] Wang J., Xu P., Zou G., Che X., Jiang X., Liu Y., Mao X., Zhang X. (2023). Integrating Spatial Transcriptomics and Single-Nucleus RNA Sequencing Reveals the Potential Therapeutic Strategies for Uterine Leiomyoma. Int. J. Biol. Sci..

[108] Luo X., Ding L., Xu J., Chegini N. (2005). Gene Expression Profiling of Leiomyoma and Myometrial Smooth Muscle Cells in Response to Transforming Growth Factor-β. Endocrinology.

[109] Serna V.A., Wu X., Qiang W., Thomas J., Blumenfeld M.L., Kurita T. (2018). Cellular Kinetics of MED12-Mutant Uterine Leiomyoma Growth and Regression in Vivo. Endocr. Relat. Cancer.

[110] Hung R.J., Terman J.R. (2011). Extracellular Inhibitors, Repellents, and Semaphorin/Plexin/MICAL-Mediated Actin Filament Disassembly. Cytoskeleton.

[111] Yang Q., Al-Hendy A. (2023). Update on the Role and Regulatory Mechanism of Extracellular Matrix in the Pathogenesis of Uterine Fibroids. Int. J. Mol. Sci..

[112] Crabtree J.S., Jelinsky S.A., Harris H.A., Choe S.E., Cotreau M.M., Kimberland M.L., Wilson E., Saraf K.A., Liu W., McCampbell A.S. (2009). Comparison of Human and Rat Uterine Leiomyomata: Identification of a Dysregulated Mammalian Target of Rapamycin Pathway. Cancer Res..

[113] Bielesz B., Sirin Y., Si H., Niranjan T., Gruenwald A., Ahn S., Kato H., Pullman J., Gessler M., Haase V.H. (2010). Epithelial Notch Signaling Regulates Interstitial Fibrosis Development in the Kidneys of Mice and Humans. J. Clin. Investig..

[114] Al-Hendy A., Diamond M.P., Boyer T.G., Halder S.K. (2016). Vitamin D3 Inhibits Wnt/β-Catenin and MTOR Signaling Pathways in Human Uterine Fibroid Cells. J. Clin. Endocrinol. Metab..

[115] Cohen M.L., Brumwell A.N., Ho T.C., Garakani K., Montas G., Leong D., Ding V.W., Golden J.A., Trinh B.N., Jablons D.M. (2024). A Fibroblast-Dependent TGFβ1/SFRP2 Noncanonical Wnt Signaling Axis Promotes Epithelial Metaplasia in Idiopathic Pulmonary Fibrosis. J. Clin. Investig..

[116] Varghese B.V., Koohestani F., McWilliams M., Colvin A., Gunewardena S., Kinsey W.H., Nowak R.A., Nothnick W.B., Chennathukuzhi V.M. (2013). Loss of the Repressor REST in Uterine Fibroids Promotes Aberrant G Protein-Coupled Receptor 10 Expression and Activates Mammalian Target of Rapamycin Pathway. Proc. Natl. Acad. Sci. USA.

[117] Ishikawa H., Ishi K., Ann Serna V., Kakazu R., Bulun S.E., Kurita T. (2010). Progesterone Is Essential for Maintenance and Growth of Uterine Leiomyoma. Endocrinology.

[118] Li Z., Yin H., Shen Y., Ren M., Xu X. (2021). The Influence of Phenolic Environmental Estrogen on the Transcriptome of Uterine Leiomyoma Cells: A Whole Transcriptome Profiling-Based Analysis. Ecotoxicol. Environ. Saf..

[119] Voronin D., Sotnikova N., Rukavishnikov K., Malyshkina A., Nagornii S., Antsiferova Y. (2021). Differential Regulatory Effect of Progesterone on the Proliferation and Apoptosis of Uterine Leiomyoma Tissue Explants and Primary Leiomyoma Cell Cultures. J. Bras. Reprod. Assist..

[120] Reschke L., Afrin S., El Sabah M., Charewycz N., Miyashita-Ishiwata M., Borahay M.A. (2022). Leptin Induces Leiomyoma Cell Proliferation and Extracellular Matrix Deposition via JAK2/STAT3 and MAPK/ERK Pathways. F&S Sci..

[121] He Y.Y., Cai B., Yang Y.X., Liu X.L., Wan X.P. (2009). Estrogenic G Protein-Coupled Receptor 30 Signaling Is Involved in Regulation of Endometrial Carcinoma by Promoting Proliferation, Invasion Potential, and Interleukin-6 Secretion via the MEK/ERK Mitogen-Activated Protein Kinase Pathway. Cancer Sci..

[122] Tal R., Segars J.H. (2014). The Role of Angiogenic Factors in Fibroid Pathogenesis: Potential Implications for Future Therapy. Hum. Reprod. Update.

[123] Afrin S., El Sabeh M., Islam M.S., Miyashita-Ishiwata M., Malik M., Catherino W.H., Akimzhanov A.M., Boehning D., Yang Q., Al-Hendy A. (2021). Simvastatin Modulates Estrogen Signaling in Uterine Leiomyoma via Regulating Receptor Palmitoylation, Trafficking and Degradation. Pharmacol. Res..

[124] Zhang Y., Cao C., Du S., Fan L., Zhang D., Wang X., He M. (2021). Estrogen Regulates Endoplasmic Reticulum Stress-Mediated Apoptosis by ERK-P65 Pathway to Promote Endometrial Angiogenesis. Reprod. Sci..

[125] Valladares F., Frías I., Báez D., García C., López F.J., Fraser J.D., Rodríguez Y., Reyes R., Díaz-Flores L., Bello A.R. (2006). Characterization of Estrogen Receptors Alpha and Beta in Uterine Leiomyoma Cells. Fertil. Steril..

[126] Li F., Wang J., Liu W. (2023). Search for Key Genes, Key Signaling Pathways, and Immune Cell Infiltration in Uterine Fibroids by Bioinformatics Analysis. Medicine.

[127] Yang R., Wang M., Zhang G., Li Y., Wang L., Cui H. (2021). POU2F2 Regulates Glycolytic Reprogramming and Glioblastoma Progression via PDPK1-Dependent Activation of PI3K/AKT/MTOR Pathway. Cell Death Dis..

[128] Zhou S., Yi T., Shen K., Zhang B., Huang F., Zhao X. (2011). Hypoxia: The Driving Force of Uterine Myometrial Stem Cells Differentiation into Leiomyoma Cells. Med. Hypotheses.

[129] Ono M., Maruyama T., Masuda H., Kajitani T., Nagashima T., Arase T., Ito M., Ohta K., Uchida H., Asada H. (2007). Side Population in Human Uterine Myometrium Displays Phenotypic and Functional Characteristics of Myometrial Stem Cells. Proc. Natl. Acad. Sci. USA.

[130] Mas A., Nair S., Laknaur A., Simón C., Diamond M.P., Al-Hendy A. (2015). Stro-1/CD44 as Putative Human Myometrial and Fibroid Stem Cell Markers. Fertil. Steril..

[131] Wu X., Serna V.A., Thomas J., Qiang W., Blumenfeld M.L., Kurita T. (2017). Subtype-Specific Tumor-Associated Fibroblasts Contribute to the Pathogenesis of Uterine Leiomyoma. Cancer Res..

[132] Buyukcelebi K., Duval A.J., Abdula F., Elkafas H., Seker-Polat F., Adli M. (2024). Integrating Leiomyoma Genetics, Epigenomics, and Single-Cell Transcriptomics Reveals Causal Genetic Variants, Genes, and Cell Types. Nat. Commun..

[133] Regner M.J., Wisniewska K., Garcia-Recio S., Thennavan A., Mendez-Giraldez R., Malladi V.S., Hawkins G., Parker J.S., Perou C.M., Bae-Jump V.L. (2021). A Multi-Omic Single-Cell Landscape of Human Gynecologic Malignancies. Mol. Cell.

[134] Hedlund E., Deng Q. (2018). Single-Cell RNA Sequencing: Technical Advancements and Biological Applications. Mol. Asp. Med..

[135] Boakye Serebour T., Cribbs A.P., Baldwin M.J., Masimirembwa C., Chikwambi Z., Kerasidou A., Snelling S.J.B. (2024). Overcoming Barriers to Single-Cell RNA Sequencing Adoption in Low- and Middle-Income Countries. Eur. J. Hum. Genet..

[136] Lähnemann D., Köster J., Szczurek E., McCarthy D.J., Hicks S.C., Robinson M.D., Vallejos C.A., Campbell K.R., Beerenwinkel N., Mahfouz A. (2020). Eleven Grand Challenges in Single-Cell Data Science. Genome Biol..

[137] Heumos L., Schaar A.C., Lance C., Litinetskaya A., Drost F., Zappia L., Lücken M.D., Strobl D.C., Henao J., Curion F. (2023). Best Practices for Single-Cell Analysis across Modalities. Nat. Rev. Genet..

[138] Van de Sande B., Lee J.S., Mutasa-Gottgens E., Naughton B., Bacon W., Manning J., Wang Y., Pollard J., Mendez M., Hill J. (2023). Applications of Single-Cell RNA Sequencing in Drug Discovery and Development. Nat. Rev. Drug Discov..

[139] Elorbany S., Berlato C., Carnevalli L.S., Maniati E., Barry S.T., Wang J., Manchanda R., Kzhyshkowska J., Balkwill F. (2024). Immunotherapy That Improves Response to Chemotherapy in High-Grade Serous Ovarian Cancer. Nat. Commun..

[140] Zhou M., Zhang Y., Song W. (2024). Single-Cell Transcriptome Analysis Identifies Subclusters and Signature with N-Glycosylation in Endometrial Cancer. Clin. Transl. Oncol..

[141] González-Martínez S., Pérez-Mies B., Cortés J., Palacios J. (2024). Single-Cell RNA Sequencing in Endometrial Cancer: Exploring the Epithelial Cells and the Microenvironment Landscape. Front. Immunol..

[142] Wang F., Yue S., Huang Q., Lei T., Li X., Wang C., Yue J., Liu C. (2024). Cellular Heterogeneity and Key Subsets of Tissue-Resident Memory T Cells in Cervical Cancer. npj Precis. Oncol..

[143] Huang R., Wang F., Zou W., Li X., Lei T., Li P., Song Y., Liu C., Yue J. (2024). Tumor Endothelium-Derived PODXL Correlates with Immunosuppressive Microenvironment and Poor Prognosis in Cervical Cancer Patients Receiving Radiotherapy or Chemoradiotherapy. Biomark. Res..

[144] Sengupta P., Dutta S., Liew F., Samrot A., Dasgupta S., Rajput M.A., Slama P., Kolesarova A., Roychoudhury S. (2024). Reproductomics: Exploring the Applications and Advancements of Computational Tools. Physiol. Res..

[145] Altmäe S., Esteban F.J., Stavreus-Evers A., Simón C., Giudice L., Lessey B.A., Horcajadas J.A., Macklon N.S., D’Hooghe T., Campoy C. (2014). Guidelines for the Design, Analysis and Interpretation of “omics” Data: Focus on Human Endometrium. Hum. Reprod. Update.

[146] Gavrilescu M., Demnerová K., Aamand J., Agathos S., Fava F. (2015). Emerging Pollutants in the Environment: Present and Future Challenges in Biomonitoring, Ecological Risks and Bioremediation. New Biotechnol..

[147] Montgomery G.W., Zondervan K.T., Nyholt D.R. (2014). The Future for Genetic Studies in Reproduction. Mol. Hum. Reprod..

[148] Yan S., Zhang G., Luo W., Xu M., Peng R., Du Z., Liu Y., Bai Z., Xiao X., Qin S. (2024). PROTAC Technology: From Drug Development to Probe Technology for Target Deconvolution. Eur. J. Med. Chem..

[149] Györfi A.H., Matei A.E., Distler J.H.W. (2018). Targeting TGF-β Signaling for the Treatment of Fibrosis. Matrix Biol..

[150] Bhat A.S., Singh N.A., Rymbai E., Birendra S., Jayaram S., Selvaraj D. (2023). Importance of Fibrosis in the Pathogenesis of Uterine Leiomyoma and the Promising Anti-Fibrotic Effects of Dipeptidyl Peptidase-4 and Fibroblast Activation Protein Inhibitors in the Treatment of Uterine Leiomyoma. Reprod. Sci..

[151] Donnez J., Stratopoulou C.A., Marie-Madeleine D. (2024). Endometriosis and Adenomyosis: Similarities and Differences. Best. Pract. Res. Clin. Obstet. Gynaecol..

[152] Li X., Li C., Zhang W., Wang Y., Qian P., Huang H. (2023). Inflammation and Aging: Signaling Pathways and Intervention Therapies. Signal Transduct. Target. Ther..

[153] Wang Z., Zhang S., Xiao Y., Zhang W., Wu S., Qin T., Yue Y., Qian W., Li L. (2020). NLRP3 Inflammasome and Inflammatory Diseases. Oxid. Med. Cell. Longev..

[154] Liu T., Zhang L., Joo D., Sun S.C. (2017). NF-ΚB Signaling in Inflammation. Signal Transduct. Target. Ther..

[155] Guo Q., Jin Y., Chen X., Ye X., Shen X., Lin M., Zeng C., Zhou T., Zhang J. (2024). NF-ΚB in Biology and Targeted Therapy: New Insights and Translational Implications. Signal Transduct. Target. Ther..

[156] Bonavina G., Taylor H.S. (2022). Endometriosis-Associated Infertility: From Pathophysiology to Tailored Treatment. Front. Endocrinol..

